# The Low-Velocity Oblique Impact Resistance of 3D-Printed Bouligand Laminates

**DOI:** 10.3390/ma19081502

**Published:** 2026-04-09

**Authors:** Shuo Wang, Yangbo Li, Xianqiang Ge, Yahui Yang, Junjie Li

**Affiliations:** 1 College of Hydraulic and Environmental Engineering, China Three Gorges University, Yichang 443002, China; wangshuo970016@163.com (S.W.);; 2 POWERCHINA BEIJING Engineering Corporation Limited, Beijing 100024, China; gexianqiang@bjy.powerchina.cn

**Keywords:** Bouligand structure, impact angle, low-speed oblique impact, 3D printing (3DP)

## Abstract

**Highlights:**

- Studied damage and energy dissipation of 3DPBLs under oblique impact.
- 15° helical configuration identified as the optimal impact-resistant design.
- Periodically rotated layers enhance resistance by increasing fracture tortuosity.
- Finite element model validated against experiments with <5% deviation.

**Abstract:**

Traditional homogeneous materials often face an inherent trade-off between strength and toughness, restricting their application in high-performance impact protection. Mechanical metamaterials overcome this fundamental limitation by integrating structure and material. The 3D-printed Bouligand laminates (3DPBLs), a type of mechanical metamaterial, are renowned for their exceptional impact resistance. While the 3DPBLs have been proven to provide superior resistance under normal impact, actual service conditions inevitably involve complex, multi-directional loading. We aimed to investigate the 3DPBLs’ oblique impact resistance here. To this purpose, samples of 3DPBLs with varying helical angles (0°, 7°, 15°, 60°, 90°) were fabricated and subjected to low-velocity drop-weight impact tests at impact angles of 0°, 30°, 45°, and 60° to evaluate their damage evolution and energy dissipation. The experimental investigation exhibited distinct temporal evolutions of contact forces, with the 15° helical configuration identified as the optimal design. Further numerical analysis using a finite element model (validated with a deviation < 10%) is conducted to simulate performance under diverse impact angles in order to validate the reasonability of the experimental investigation. Mechanistically, 3DPBLs enhance impact resistance by increasing fracture tortuosity through their periodically rotated layered structure. These findings establish a theoretical foundation for developing high-performance, lightweight, and toughened protective materials.

## 1. Introduction

Impact resistance is a key performance metric for advanced protective materials across aerospace [1], transportation [2], defense [3], and civil engineering [4,5]. It is fundamentally governed by toughness, a property requiring the synergy of high strength and plasticity. While low strength causes failure through excessive deformation, poor plasticity leads to brittle fracture and limited energy dissipation. Therefore, it is essential to achieve an optimal balance between strength and plasticity. However, conventional homogeneous materials are restricted by the pervasive “strength-ductility trade-off” [6]. To address these limitations, this study adopts the paradigm of mechanical metamaterials—a design strategy that integrates material properties with artificial microstructures to transcend the inherent performance constraints of traditional materials. Mechanical metamaterials can be classified based on the properties regulated by their structural topology, primarily including negative thermal expansion [7], lightweight ultra-strong [8], tunable Young’s modulus [9], negative Poisson’s ratio (auxetic) [10], vanishing shear modulus (pentamode) [11], and negative compressibility structures [12]. Specifically, this study constructed a lightweight, high-strength mechanical metamaterial by integrating periodic helical artificial microstructures—Bouligand structures—with the matrix material. Leveraging the geometric properties of the Bouligand structures, the proposed design effectively induces crack twisting and deflection during propagation, thereby providing a reliable strategy for enhancing impact resistance [13].

The Bouligand structure adopted in this study draws inspiration from the dactyl club of the mantis shrimp [14]. This structure is renowned for its ability to dissipate stress through crack-twisting mechanisms, enabling it to withstand impact accelerations as high as 104 m/s^2^ [15]. Therefore, integrating Bouligand structures into fiber-reinforced composites can significantly enhance the overall impact resistance of the assembly. For instance, Shang et al. [16] reported that a 19-ply helicoidal laminate with a 10° pitch angle achieved a 34% increase in peak load. Similarly, investigations by Grunenfelder [17], Ginzburg [18] and Jiang [19] demonstrated that helicoidal carbon fiber-reinforced polymer (CFRP) laminates exhibited reduced through-thickness damage and enhanced residual strength under low-velocity impact. Collectively, these findings validate the Bouligand structure’s exceptional crack deflection capabilities and damage tolerance under both quasi-static and dynamic loading conditions.

Although fabricating such complex geometries was previously challenging, the maturation of fused deposition modeling (FDM) technology has enabled their precise manufacturing, opening new avenues for developing high-performance protective composites. For instance, Yang et al. [20] drew inspiration from the leaves of the Chinese scholar tree to fabricate superhydrophobic micro-scale egg beaters using an immersion surface deposition 3D printing technique. Incorporating multi-walled carbon nanotubes into the photopolymer resin enhanced the surface roughness and mechanical strength of the microstructures. Similarly, Guan et al. [21] employed Direct Ink Writing (DIW) to fabricate Bouligand-structured composites mimicking the dactyl club of a mantis shrimp. Structures with varying helix angles were tested, revealing that the Bouligand structure with a 40° helix angle exhibited the highest energy absorption capacity (2.4 kJ/m^2^). Compared to traditional unidirectional fiber materials, its impact performance improved by 140%. Guan et al. [22] also found that the Bouligand structure achieved a maximum compressive stress of 117 MPa and an energy absorption of 19 MJ/m^3^ at a pitch angle of 40°; in contrast, for the porous lattice structures, the Bouligand structure exhibited superior performance at a 90° angle. By elucidating enhancement mechanisms such as crack twisting and bridging, this study demonstrates that Bouligand structure is an effective strategy for developing lightweight, high-strength materials for aerospace and defense applications.

Materials frequently endure complex impact scenarios during service, such as rockfall impacts causing dam failures [23] and railway tracks suffering damage from falling ice [24]. Various materials, including metals [25,26,27] and composites [28,29], are susceptible to such impact loads. In real-world applications, these impacts typically occur at oblique angles rather than normal incidence. Consequently, investigating damage patterns and energy dissipation mechanisms under varying impact angles provides a more accurate reflection of actual service conditions. Indeed, the angle of incidence exerts a substantial influence on the mechanical response, damage mechanisms, and failure modes of materials and structures [30,31].

Regarding oblique impact research, Zhang et al. [32] developed a low-velocity impact simulation model for double-sided sanded laminated panels at angles ranging from 30° to 90°. Their findings demonstrated that the residual kinetic energy of the impactor was lower during 60° oblique impacts compared to vertical impacts, substantiating the hypothesis that materials absorb more energy during oblique loading. Xie et al. [33] conducted multi-angle impact tests on aluminum honeycomb sandwich panels, thereby clarifying that as the impact angle increases to 30°, the peak contact force begins to decrease, total absorbed energy diminishes, and the surface dent position shifts more significantly with increasing horizontal velocity of the impactor. Chen et al. [34] conducted numerical simulations of low-velocity impacts on carbon fiber-reinforced epoxy laminates at angles of 90°, 75°, 60°, 45°, 30°, and 15°. The findings demonstrated that rebound velocity remained constant for inclination angles between 15° and 45°, while it increased for angles between 45° and 75°. He et al. [35] conducted numerical simulations on prestressed shell structures with single-layer plates subjected to high-speed oblique impacts by conical projectiles. The investigation revealed that the crack size in the laminate increased with the incident angle.

While Bouligand structures have demonstrated significant potential in carbon fiber-reinforced polymers, their application in reinforcing inherently brittle matrices, such as cementitious composites, remains an emerging frontier. Elucidating the synergistic mechanisms between brittle matrices and bionic helical architectures is essential for transitioning these bio-inspired designs into large-scale civil engineering infrastructures. Our research team has established a robust foundation in the development and functionalization of 3D-printed concrete (3DPC). On one hand, we optimized the mix proportion of 3D-printed phosphogypsum concrete (3DPPGC) to achieve superior mechanical properties suitable for high-value engineering applications [36]. On the other hand, we developed an advanced self-sensing concrete system by embedding distributed optical fibers during the 3D printing process, enabling precise, real-time structural health monitoring [37].

Recent studies have confirmed that oblique impacts induce distinct mechanical responses, damage patterns, and energy absorption behaviors compared to normal impacts. Understanding the mechanical behavior of 3DPBLs under varying impact angles is therefore essential for accurate damage prediction and structural optimization. However, the specific response characteristics and governing parameters of 3DPBLs under oblique loading remain largely unexplored. To address this gap, this study systematically evaluates the mechanical response, damage mechanisms, and energy dissipation of a3DPBL under various impact angles via drop-weight tests and numerical simulations. This paper elucidates how the unique rotational layering of the 3DPBL mitigates through-thickness penetration during oblique impacts, providing theoretical insights for novel protective designs.

## 2. Materials and Design of Experiments

### 2.1. Test Materials and Forming Technology

#### 2.1.1. Test Materials

The raw material used in the experiment was PLA, which exhibits good thermal stability. Its typical processing temperature ranges from approximately 195 °C to 230 °C, achieving a stable molten state at 220 °C with superior plasticity and processability. At this temperature, the material demonstrates good plasticity, strong processability, and excellent mechanical properties.

We used PLA filament rolls manufactured by Shenzhen Aurora Technology Co., Ltd. (Shenzhen, China), production batch number NPLA001. Each roll of PLA filament is 340 m long with a diameter of 1.75 ± 0.05 mm. Its density is 1220 kg/m^3^, with a melt flow rate maintained at 0.78 g/min. The tensile strength is 62 MPa, tensile strain is 4.43%, flexural strength is 65 MPa, elastic modulus is 3.6 GPa, and notched impact strength is 4.28 kJ/m^2^.

#### 2.1.2. Method for Forming Bouligand Structures

**Figure 1:**
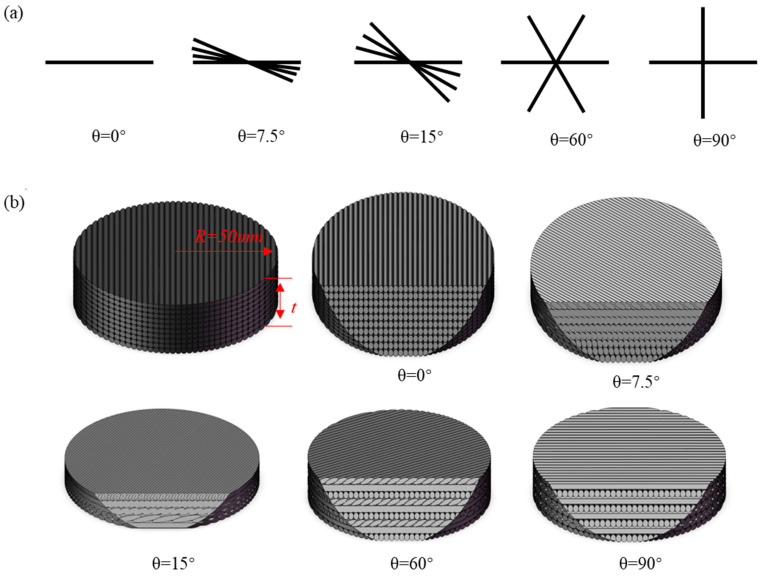
Schematic of the 3DPBL structure configurations. *R* and *t* denote the radius and the total thickness of the specimen, respectively. *θ* represents the helical pitch angle (inter-layer rotation angle). (**a**) Planar printing path orientations for different layers. (**b**) Cross-sectional views at various helical angles.

**Figure A1:**
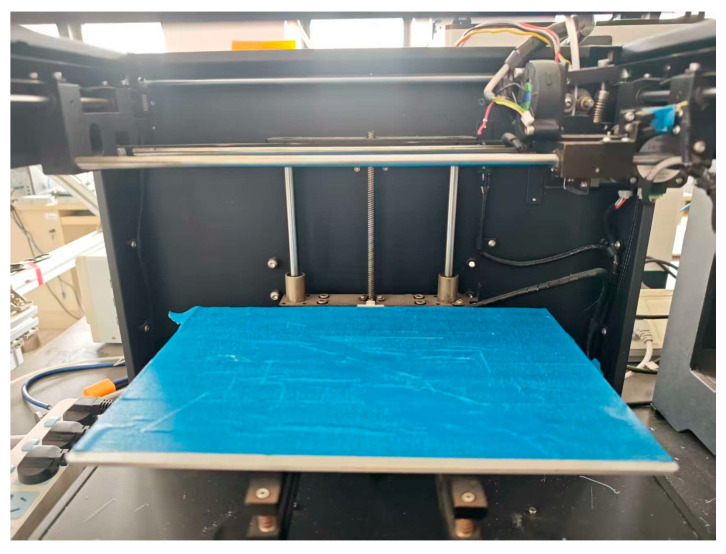
FDM 3D printer.

Test specimens of the 3DPBL were printed using PLA filament on a custom-built FDM 3D printer developed by the laboratory at China Three Gorges University (Yichang, China) (Figure A1). The 3DPBL was designed with a tilt angle that progressively increases with each layer based on a specific angular increment, as shown in Figure 1a. To realize this design, the scripting language of the Cura 4.13.1 slicing software (Ultimaker B.V., Utrecht, The Netherlands) was modified. A for-loop was integrated into the base code for rectilinear infill to iteratively extract and update the deposition angle variable for each layer, thus enabling a helical ascent angle. Compared to direct modification of G-code coordinates, this slicer-based approach reduced subsequent processing effort and introduced a novel infill pattern. The printer then fabricated 3DPBLs with varying helical angles by following these modified toolpaths, as demonstrated in Figure 1b.

### 2.2. Mechanical Properties Testing

**Figure A2:**
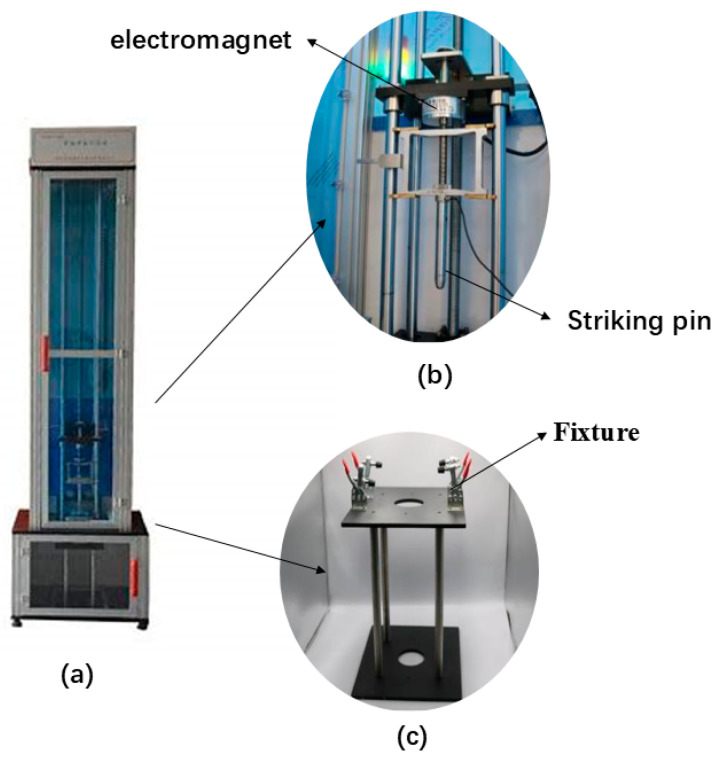
Drop-weight impact test platform. (**a**) Drop-weight impact testing apparatus. (**b**) Drop-weight impact testing machine indenter. (**c**) Specimen clamping platform.

Drop-weight impact tests were conducted in accordance with the ISO 179-1-2023 [38] test standard and the ASTM D7136 [39] test specification. The tests were performed using CLC-AI drop-weight impact testing apparatus (Beijing Guancai Precision Instrument Co., Ltd., Beijing, China), as shown in Figure A2.

**Figure 2:**
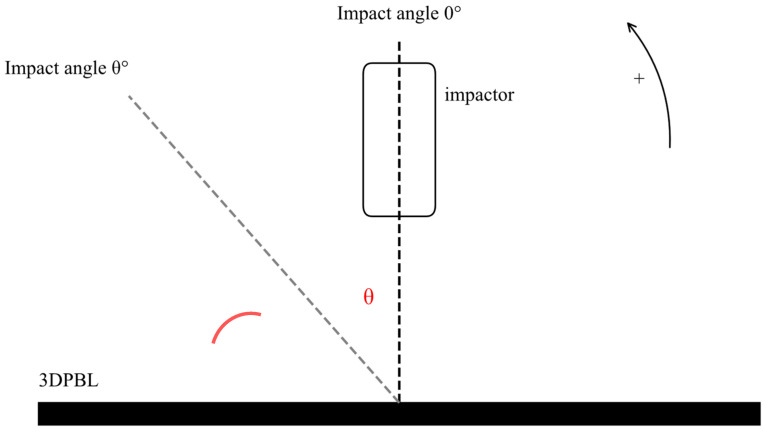
Definition of the oblique impact angle (θ) in the experimental setup. The curved arrow on the upper right side, along with the “+” sign, denotes that the counter-clockwise rotation is defined as the positive direction for the impact angle θ.

**Figure 3:**
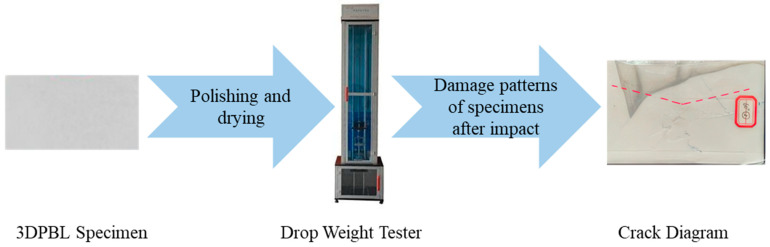
Schematic diagram of the experimental process: from specimen preparation to damage analysis. The red dashed line indicates the crack propagation path.

The impact angle of the 3DPBL is defined as the angle between the impactor axis and the normal to the specimen plane, as illustrated in Figure 2. In accordance with the provisions stipulated in ASTM D7136 [39], the dimensions of the specimen are specified as 150 mm × 100 mm × 4 mm for the 3DPBL. The flowchart for the drop hammer impact test is shown in Figure 3. The theoretical thickness of the single layer is 0.4 mm, with 10 layers printed using Fused Deposition Modeling (FDM). The specific parameters of the PLA 3DPBL produced by the 3D printing equipment are detailed in Table 1.

**Figure 4:**
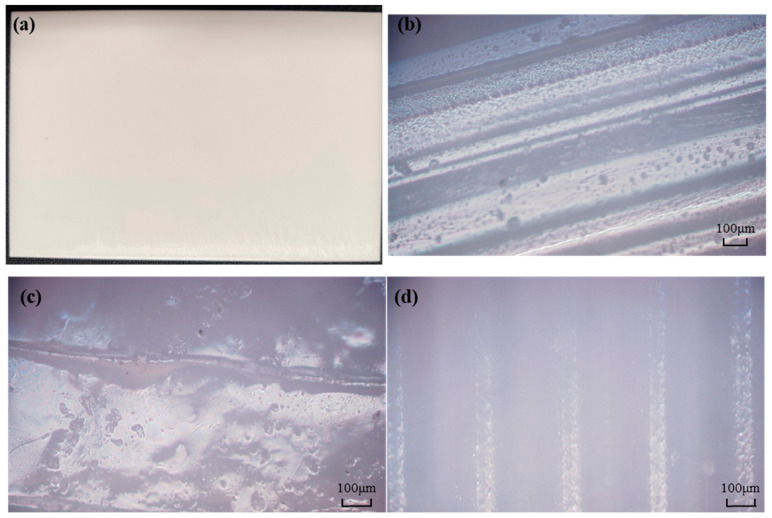
Morphological observations of the pristine 3D-printed PLA specimen: (**a**) Macroscopic view demonstrating the general shape and high-quality surface finish; (**b**) Microscopic view of the front surface showing uniform and continuous printing paths; (**c**) Microscopic view of the side surface illustrating dense layer-by-layer stacking and robust interlaminar bonding; (**d**) Microscopic view of the back surface displaying consistent material fusion without notable macroscopic voids.

Prior to impact testing, the general appearance and microscopic morphology of the pristine PLA specimens were carefully inspected to verify their manufacturing quality and structural integrity (Figure 4). As shown in Figure 4a, the macroscopic view demonstrates high dimensional accuracy, a flat surface finish, and the absence of macroscopic defects such as warping. Further microscopic observations corroborate this high printing quality. The microscopic images of the front and back surfaces (Figure 4b and Figure 4d), respectively display uniform, parallel printing paths with robust material fusion and no noticeable macroscopic voids or severe under-extrusion. Additionally, the cross-sectional microscopic view from the side (Figure 4c) reveals a highly dense layer-by-layer stacking, indicating excellent interlaminar bonding.

The apparatus used for this study was a semicircular impactor with a diameter of 16 mm and a counterweight mass of 2.4 kg. The impact velocity of the apparatus was 1.44 m/s, and the impact energy delivered was 2.5 J.

To ensure the reproducibility and reliability of the experimental findings, triplicate independent specimens were fabricated and tested for each configuration (encompassing various helix and impact angles). The mechanical metrics reported herein represent the arithmetic mean of these three measurements. This sample size (*n* = 3) was selected owing to the high manufacturing precision and structural consistency inherent in the FDM process, which typically yields minimal intra-group variability. Preliminary tests confirmed that three repetitions are sufficient to capture the characteristic impact response and provide a statistically sound representation of the structural performance while maintaining experimental efficiency.

## 3. Numerical Simulation

### 3.1. Constitutive Model

The 3DPBL is formed by the spiral stacking of identical straight filaments, with each layer serving as its fundamental unit. The 3DPBL, fabricated via the FDM method, exhibits distinct mechanical anisotropy.

**Figure 5:**
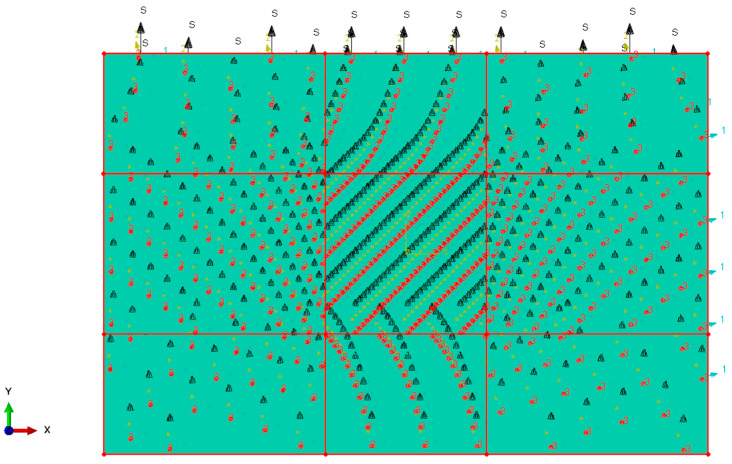
Schematic of the layer-by-layer modeling approach and the assignment of local material orientations based on 3D printing raster paths. The black arrows denote the local material orientations defined for each individual layer. The blue, yellow, and red arrows represent the global $X, Y$, and $Z$ axes of the coordinate system, respectively.

The 3DPBL was numerically modeled using a discrete layer-by-layer approach to capture the intrinsic directional dependency of the FDM process. Crucially, the material orientation was defined layer-by-layer to ensure that the local principal axes were consistently aligned with the 3D printing raster paths, as illustrated in Figure 5.

In order to analyze the cumulative damage caused by impact to the 3DPBL, it is necessary to introduce a damage model. The present study employs the Hashin criterion, a linear evolution method, and zero-thickness cohesive elements for simulation.

(1) $$\sigma_{ij}=C_{ijkl}\varepsilon_{kl}$$

In the equation, *σ*_*ij*_ denotes stress, *C*_ij*kl*_ represents the stiffness matrix, and *ε*_*kl*_ indicates the strain component [40]. Moreover, in consideration of the transverse isotropy exhibited by single-layer structures.

The damage stiffness matrix [41] is a symmetric matrix represented by Equation (2). In this equation, *d*_*Xt*_ denotes the tensile loss variable in the *X* direction, *d*_*Yt*_ denotes the tensile loss variable in the *Y* direction, and *d*_*Xc*_ denotes the compressive loss variable in the *X* direction. The symbol *d*_*Yc*_ denotes the tensile damage variable in the *Y* direction, *S*_*Yt*_ represents the stiffness correction coefficient for shear strength loss due to tensile damage in the *Y* direction, and *S*_*Yc*_ denotes the stiffness correction coefficient for shear strength loss due to compressive damage in the *Y* direction. The relationships between these variables are specified in Equations (2) and (3).

(2) $$c^{d}=\frac{1}{\Delta }\left[\begin{array}{ccccc} d_{f}E_{11}(1-d_{m}v_{23}v_{32}) & d_{f}d_{m}E_{11}(v_{21}-v_{23}v_{31}) & d_{f}E_{11}(v_{31}-d_{m}v_{21}v_{32}) & & \\ & d_{m}E_{22}(1-d_{f}v_{13}v_{31}) & d_{m}E_{22}(v_{32}-d_{f}v_{12}v_{31}) & & \\ & & E_{33}(1-d_{f}d_{m}v_{12}v_{21}) & \Delta d_{f}d_{m}G_{12} & \\ & & & \Delta d_{f}d_{m}G_{23} & \\ & & & & \Delta d_{f}d_{m}G_{13} \end{array}\right]$$

(3) $$\left\{\begin{array}{l} d_{f}=(1-d_{ft})(1-d_{fc})\\ d_{m}=(1-S_{mt}d_{mt})(1-S_{mc}d_{mc})\\ \Delta =1-d_{f}d_{m}v_{12}v_{21}-d_{m}v_{23}v_{32}-d_{f}v_{13}v_{31}-2d_{f}d_{m}v_{21}v_{32}v_{13} \end{array}\right.$$

In consideration of the structural disparities inherent in this paper, and with a view to aligning with the compositional logic of the model, the damage initiation criteria have undergone modification from fiber damage and matrix damage to in-fiber damage and transverse damage. The initiation of in-fiber damage is predicted using the maximum stress criterion, as expressed by Equation (4).

(4) $$\left\{\begin{array}{cc} F_{ft}={\left(\frac{\sigma_{11}}{X^{T}}\right)}^{2}, & \left(\sigma_{11}>0\right)\\ F_{fc}={\left(\frac{\sigma_{11}}{X^{C}}\right)}^{2}, & \left(\sigma_{11}<0\right) \end{array}\right.$$

*F*_ft_ and *F*_fc_ are used to denote the failure criteria for tensile and compressive stress in the X direction, respectively, while *X*^T^ and *X*^C^ represent the tensile and compressive strengths in the fiber direction.

Common damage initiation criteria are the Hashin criterion and the Puck criterion [42,43,44,45]. In consideration of the mechanical similarity between the research model and fiber-reinforced composite laminates, the Hashin criterion is adopted for the matrix damage initiation criterion, as demonstrated in Equation (5).

(5) $$\begin{array}{c} F_{mt}={\left(\frac{\sigma_{22}+\sigma_{33}}{Y^{T}}\right)}^{2}+\frac{1}{S_{23}^{2}}(\sigma_{23}^{2}-\sigma_{22}\sigma_{33})+{\left(\frac{\sigma_{12}}{S_{12}}\right)}^{2}+{\left(\frac{\sigma_{13}}{S_{13}}\right)}^{2}\geq 1,\\ \left(\sigma_{22}+\sigma_{33}>0\right)\\ F_{mc}={\left(\frac{\sigma_{22}+\sigma_{33}}{2S_{23}}\right)}^{2}+\frac{\sigma_{22}+\sigma_{33}}{Y^{C}}\left[{\left(\frac{Y^{C}}{2S_{23}}\right)}^{2}-1\right]+\frac{1}{S_{23}^{2}}(\sigma_{23}^{2}-\sigma_{22}\sigma_{33})\\ +{\left(\frac{\sigma_{12}}{S_{12}}\right)}^{2}+{\left(\frac{\sigma_{13}}{S_{13}}\right)}^{2}\geq 1,(\sigma_{22}+\sigma_{33}<0) \end{array}$$

It is only when the damage initiation criteria are satisfied that the damage evolution method becomes effective.

### 3.2. Cohesive Element

**Figure 6:**
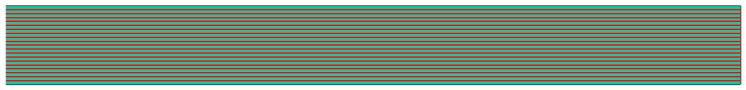
Detailed view of the zero-thickness cohesive elements (highlighted in red) inserted between discrete layers.

The phenomenon of layered damage has been identified as the primary cause of material collapse in laminated composites. It is considered the most critical failure mode in this context, as it propagates in an undetectable and unpredictable manner. In order to fully account for the effects of delamination damage, this study incorporates zero-thickness cohesive elements with bilinear constitutive behavior when establishing the simulation model. The implementation and placement of these cohesive elements at the interlaminar interfaces are illustrated in Figure 6, where the red layers represent the zero-thickness regions.

In the process of assigning computational properties to the elements, the quadratic failure criterion (Formula (6)) from the interlaminar bilinear cohesive model for composite materials under mixed-mode loading proposed by Camanho et al. [46] and the B-K law (Formula (7)) [47] are employed to predict the initiation and propagation of delamination damage.

(6) $${\left(\frac{t_{n}}{N}\right)}^{2}+{\left(\frac{t_{s}}{S}\right)}^{2}+{\left(\frac{t_{t}}{T}\right)}^{2}=1$$

The symbol *tn* denotes the n-direction traction force, *ts* denotes the s-direction traction force, and *tt* denotes the t-direction traction force; *N* denotes the n-direction normal strength, *S* denotes the s-direction normal strength, and *T* denotes the shear strength.

(7) $$G_{c}=G_{IC}+(G_{IIC}-G_{IC}){\left\{\frac{G_{shear}}{G_{T}}\right\}}^{n}$$

The acronyms *G*_C_, *G*_IC_ and *G*_IIC_ represent the total fracture energy, the normal fracture energy and the shear critical fracture energy, respectively. The symbols *G*_*shear*_ and *G*_*T*_ represent dissipated energy in the out-of-plane direction and total dissipated energy in all three directions, respectively. The material coefficient in the B-K law, typically denoted by *η*, is represented by the symbol *η*, which is equal to 1.45 [48,49].

It is evident that, due to the inherent properties of brittle materials, PLA primarily exhibits a hybrid damage pattern, combining interlaminar pitting damage with plane-penetrating cracks. Preliminary mechanical analysis suggests that crack propagation may exhibit a simpler propagation pattern when compared to interlaminar damage. Consequently, the failure criterion employs the maximum stress criterion (Formula (8)).

(8) $$\mathrm{MAX}\left\{\frac{\langle t_{n}\rangle }{N},\frac{\langle t_{s}\rangle }{S},\frac{\langle t_{t}\rangle }{T}\right\}=1$$

## 4. Results and Discussion

### 4.1. Experimental and Numerical Study on Low-Velocity Normal Impact of the 3DPBL

**Figure 7:**
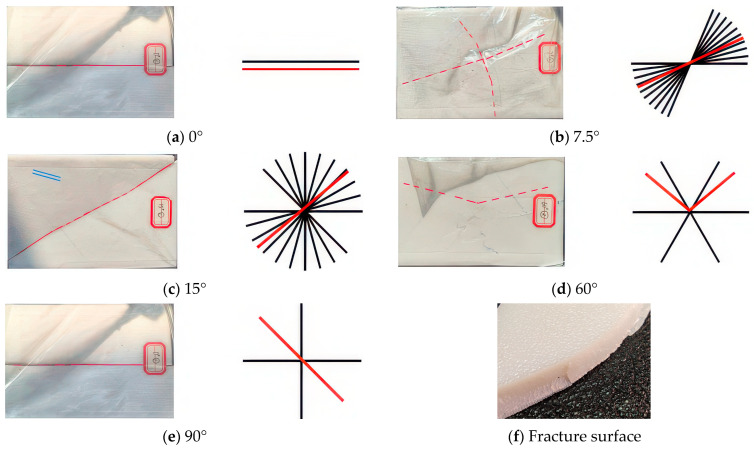
Damage patterns of 3DPBL specimens subjected to impact at different helical angles (θ): (**a**) 0°, (**b**) 7.5°, (**c**) 15°, (**d**) 60°, (**e**) 90°, and (**f**) the fracture surface. The red lines denote the actual splitting locations, whereas the black lines define the stacking orientations of the helical structure.

A thorough post-impact examination of the 3DPBL specimens reveals that damage primarily manifests as through-thickness cracks and localized indentations (Figure 7). As illustrated in Figure 7, fracture patterns vary significantly depending on the helix angle. At a 0° angle, a single crack appears parallel to the fiber direction and centered on the plate. As the helix angle increases from 5° to 15°, the damage transitions into cross-shaped through-cracks; notably, at 15°, these cracks align with the diagonals of the specimen. In contrast, the 60° specimen exhibits the most distinctive fracture pattern, characterized by divergent cracks radiating from the center with inter-crack angles of approximately 120°. This is the only configuration where zigzag-shaped through-cracks are observed.

Furthermore, all cracks tend to propagate along the primary fiber orientation, a phenomenon attributed to the structural weak planes inherent in the fiber deposition paths. To further verify the structural integrity and fabrication quality that govern these failure modes, the cross-sectional morphology of the as-printed specimen was examined (Figure 7f). The high-resolution photograph in Figure 7f reveals a highly dense internal structure with negligible macro-scale voids, confirming superior fusion between the deposited PLA filaments. The clearly discernible layers exhibit consistent thickness, aligning with the prescribed 0.4 mm parameters. Under impact loading, the impacted surface undergoes compressive indentation, while the rear surface experiences tensile stress analogous to a simply supported beam, leading to preferential failure along these weak structural planes. This analysis clarifies the characteristic crack locations and their underlying formation mechanisms.

In order to conduct a more comprehensive and multidimensional analysis of energy conversion during the impact process, Equations (9)–(11) specified in the ASTM D7136 standard were applied to calculate the contact force *F*(*t*) and velocity *V_i_*. This process yielded the time-history curve of the impactor velocity variation and absorbed energy.

This process yielded the time-history curves for impactor velocity variation and energy absorption. To ensure a representative visualization of the dynamic response, the contact force data *F*(*t*) and the subsequent calculated results presented herein were selected from the test specimen that most closely aligned with the arithmetic mean of the triplicate measurements for each configuration. This selection criterion ensures that the reported time-history curves accurately reflect the typical mechanical behavior within each group.

(9) $$v(t)=v_{i}+gt-\int_{0}^{t}\frac{F(t)}{m}dt\;$$

(10) $$\delta (t)=\delta_{i}+v_{i}t+\frac{gt^{2}}{2}-\int_{0}^{t}\left(\int_{0}^{t}\frac{F(t)}{m}dt\right)dt\;$$

(11) $$E_{a}(t)=\frac{m(v_{i}^{2}-v{\left(t\right)}^{2})}{2}+mg\delta (t)\;$$

The peak contact forces, along with their corresponding mean values and standard deviations for the three specimens in each test configuration, are summarized in Table 2.

**Figure 8:**
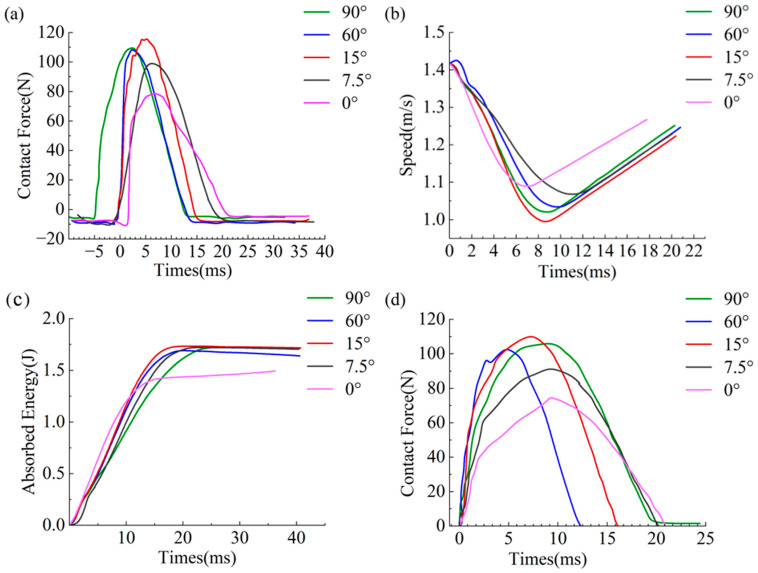
Time history curves of the impact response. (**a**) Contact force F versus time *T* during impact testing. (**b**) Velocity v versus time *T* during impact testing. (**c**) Absorbed energy Ea versus time *T* during impact testing. (**d**) Numerically simulated contact force F versus time *T* curve.

Figure 8a–c shows the curve illustrating the contact force between the impactor and the 3DPBL specimen over time during the impact process. The peak impact force at the moment of impact failure was extracted, with peak contact forces corresponding to helix angles of 0°, 7.5°, 15°, 60°, and 90° being 79.8 N, 99.86 N, 116.49 N, 109.19 N, and 110.22 N, respectively.

In summary, analysis of the temporal variations in contact force, velocity, and absorbed energy reveals that these variables represent the deformation resistance of the 3DPBL with different helix angles under a 2.5 J impact. The findings of the present study demonstrate that the mechanical properties of the 3DPBL are optimal at a helix angle of 15°, while the unhelix (0°) 3DPBL exhibits the poorest mechanical performance.

### 4.2. Multi-Angle Low-Speed Impact Simulation Study of the 3DPBL

#### 4.2.1. Establishment of Multi-Angle Impact Simulation Models

**Figure A3:**
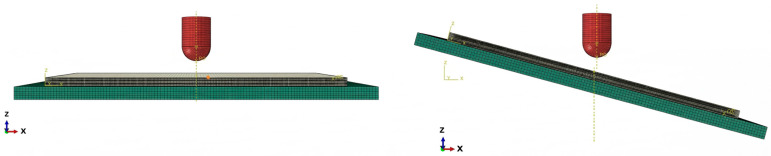
Multi-angle impact simulation model.

The multi-angle impact simulation model, as illustrated in Figure A3, consists of four primary components: the impactor, the fixture base, the 3DPBL specimen, and the zero-thickness cohesive elements. The hemispherical impactor is modeled using R3D4 discrete rigid elements, while the fixture base is meshed with C3D8R eight-node linear brick elements. To accurately replicate the 3D-printed architecture, the 3DPBL was subdivided into ten discrete layers through geometry partitioning, with each layer meshed as a single stack of C3D8R solid elements with a thickness of 0.4 mm. This partitioning strategy ensures that the interlaminar interfaces align perfectly for the subsequent insertion of COH3D8 cohesive elements.

The effects of interlayer delamination damage were simulated by inserting COH3D8 elements between adjacent specimen layers, with material parameters as shown in Table 3. The impactor impact velocity was calculated using the standard impact energy-velocity equation, Equation (12), defining the impact velocity as *v_i_* = 1.44 m/s.

(12) $$E_{i}=\frac{mv_{i}^{2}}{2}$$

**Figure A4:**
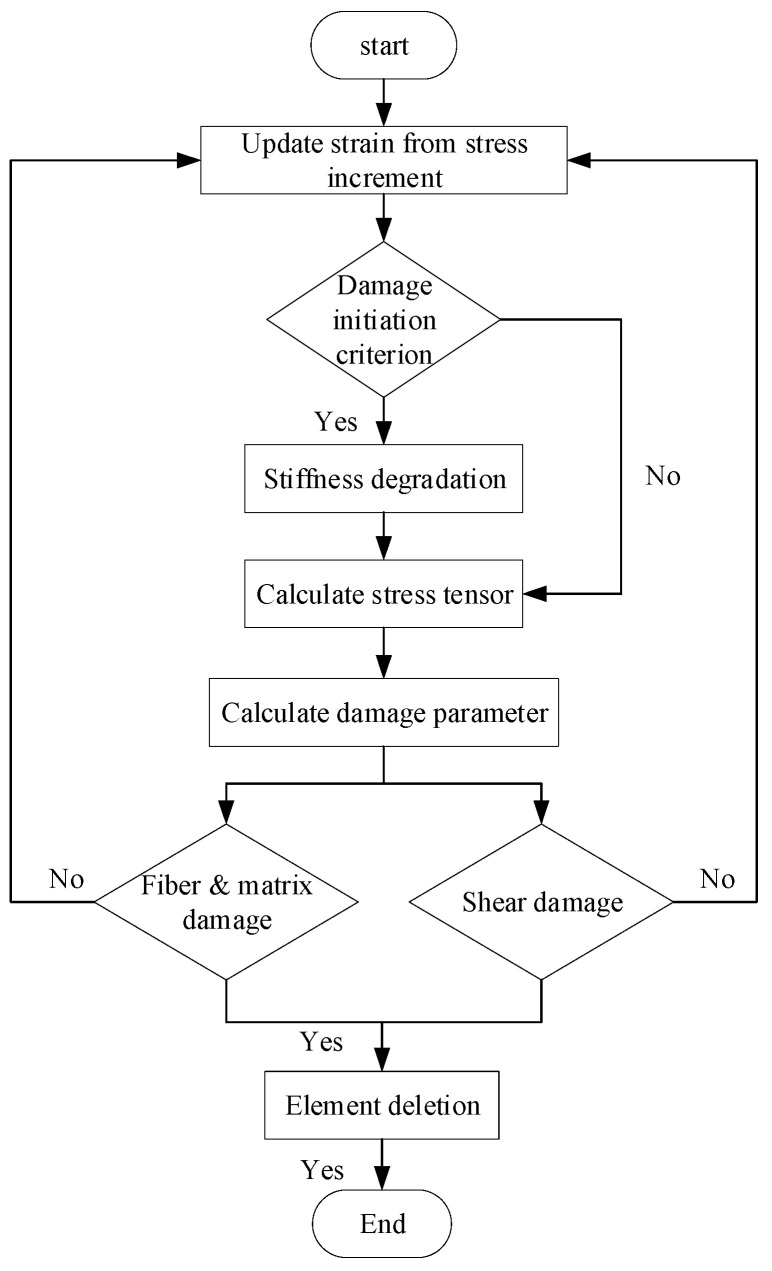
VUMAT’s operational logic.

The properties of the specimen model were assigned on the basis of the constitutive and damage models for the PLA that were deemed to be relevant. Solid elements were utilized in the construction of the 3DPBL, with the Hashin criterion being adopted as the damage initiation criterion. Consequently, the VUMAT subroutine must be incorporated into the final calculations. The program logic is illustrated in Figure A4.

#### 4.2.2. Multi-Angle Impact Simulation Model Validation

To ensure the convergence and accuracy of the numerical results, a mesh sensitivity analysis was initially conducted by varying the in-plane element size of the 3DPBL from 0.5 mm to 2.0 mm. Considering the theoretical layer thickness of 0.4 mm, the peak contact force and total energy absorption were selected as key convergence indicators. The results stabilized when the in-plane mesh size reached 0.8 mm, with further refinement yielding a negligible deviation (less than 2.5%). Consequently, a mesh configuration with a 0.8 mm in-plane size and a 0.4 mm through-thickness size was adopted to balance computational efficiency and capture the steep stress gradients within the helical architecture.

The interfaces between the impactor, the 3DPBL and the fixture should be set as “face-to-face contact properties”. The “hard” contact model should be applied for normal contact, with negative material gaps being disallowed. A zero gap indicates full contact, allowing constraints or loads to be applied to assist simulation. In order to facilitate tangential contact, it is necessary to employ the “penalty” function model, with a motion damping coefficient of 0.3. In accordance with the principles of the Coulomb sliding friction model, it has been demonstrated that the magnitude of the frictional force is directly proportional to the contact pressure. However, in three-dimensional simulations, the shear stress between solid interfaces consists of two orthogonal components. For the purpose of calculation, it is necessary to represent these components by means of equivalent shear stress, as expressed in Formula (13).

(13) $$\tau =\sqrt{\tau_{1}^{2}+\tau_{2}^{2}}$$

The impactor constraint should be set to retain only the Z-direction translational degree of freedom; the fixture base should be defined as a rigid body with fully fixed boundary conditions applied; boundary conditions must be established via hinged connections (U1 = U2 = U3) on the specimen sides to ensure structural integrity. Forward low-speed impact simulations must be conducted under these conditions. Subsequent steps involve plotting time-dependent contact force curves for both experimental and simulated results from drop hammer tests on the 3DPBL with varying helix angles (Figure 8a,d). Peak contact force data are extracted (Table 4), and relative error margins between experimental and simulated values are calculated. The results demonstrate that the overall trends of the simulation are in good agreement with the experimental data, with the relative error consistently maintained within 10%. Consequently, the simulation model is considered reliable, providing a robust basis for subsequent analysis of oblique impact damage.

#### 4.2.3. Simulation Analysis of Finite Element Results at Different Impact Angles

In comparison with the forward impact process, during low-speed inclined impacts, the impactor makes contact with the 3DPBL earlier. Throughout the course of the continuous downward pressure, a certain degree of asymmetry is exhibited in the contact deformation between the two. Furthermore, due to the persistent angle between the impactor and the 3DPBL surface, the impact force exhibits both normal and tangential components relative to the 3DPBL surface. The concurrent occurrence of tangential sliding and normal loading during the loading process is attributable to the aforementioned component forces, with a proportion of the impactor’s kinetic energy being dissipated through sliding friction. In order to prevent errors in the modeling process during the simulation of inclined impact loads, which differ from rebound processes, it is necessary to vary the contact properties between the impact conditions. In the case of inclined impacts, it is necessary to alter the contact type between the impactor and the 3DPBL surface from “surface-to-surface contact” to “general contact”.

This study investigates the damage characteristics and mechanical responses of 3DPBLs under constant oblique impact angles, with the helix angle serving as the primary experimental variable. The experimental plan is detailed in Table 5.

**Figure 9:**
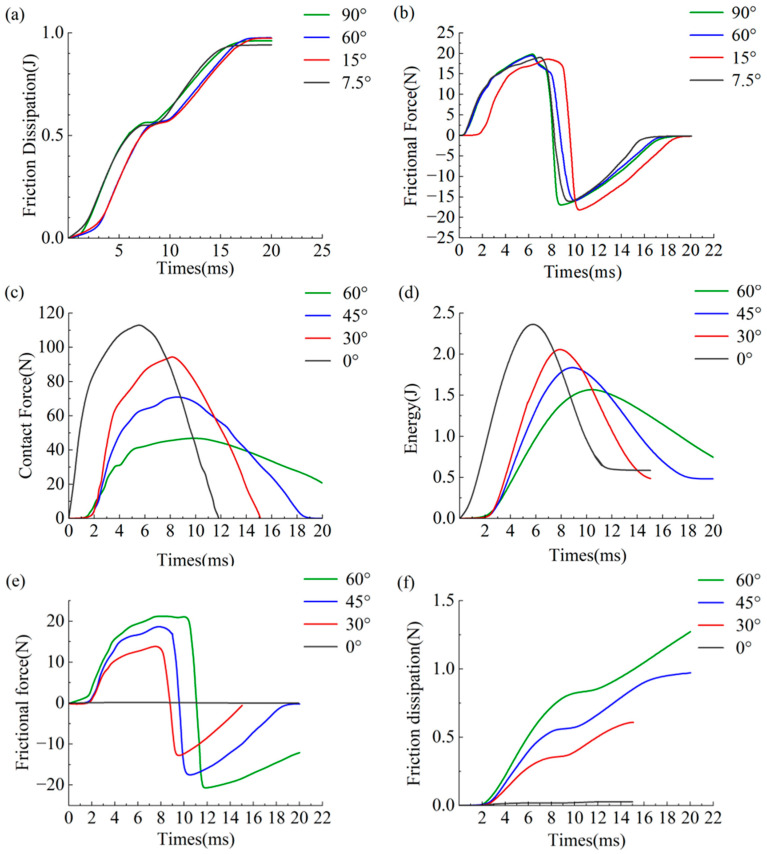
Time histories of the mechanical response for 3DPBLs. (**a**,**b**) Effect of helix angle on (**a**) friction energy dissipation ∆E versus time *T* and (**b**) friction force Ff versus time t. (**c**–**f**) Effect of impact angle on (**c**) contact force F versus time t, (**d**) internal energy E versus time t, (**e**) frictional force Ff versus time t, and (**f**) frictional energy dissipation ∆E versus time t.

Figure 9a illustrates the frictional energy dissipation for 3DPBLs at helix angles of 7.5°, 15°, 60°, and 90°, with all values approximating 1 J. In Figure 9b, negative values denote the loading phase of the impact. As the impactor’s kinetic energy reaches zero, the stored strain energy within the 3DPBL is released, initiating the rebound. During this transition, the friction force reverses to positive, while its peak magnitude remains essentially unchanged.

**Figure 10:**
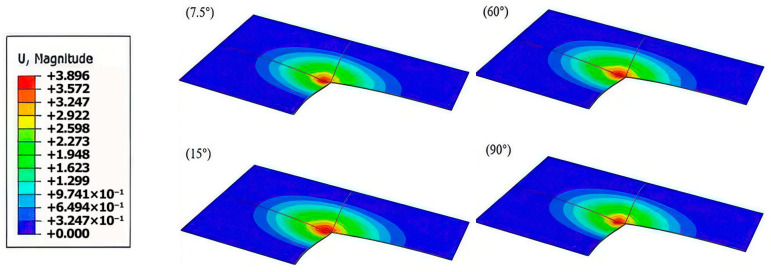
Cross-sectional deformation contours of 3DPBL specimens with different helix angles.

A comparison of the damage patterns across 3DPBLs with varying helix angles under identical oblique impact conditions Figure 10 reveals an inverse relationship between the maximum indentation depth and the impact resistance of the structure. Furthermore, observations indicate that for all five helix angles studied, the tangential displacement of the impactor scales positively with the impact angle under a constant impact energy.

**Figure 11:**
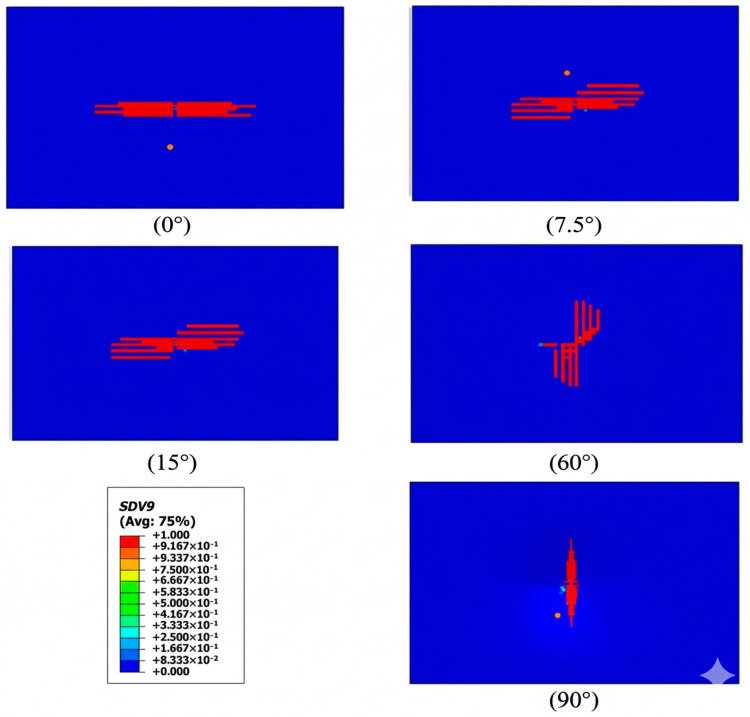
Crack propagation characteristics under different helical angles.

In addition to indentations, the 3DPBL also exhibits crack damage under impact loading. A detailed investigation into crack failure units across a range of helix angle structures has revealed that the initiation of cracks is primarily caused by tensile stresses induced by impact loading at the base, acting on inter-thread weak planes. The fundamental cause of this damage is attributed to 3DPBL normal loading. Consequently, as the helix angle varies, the crack location exhibits a similar trend to that observed in normal impact (Figure 11). However, due to the reduced normal load component, the damage depth in an inclined impact is smaller compared to a normal (0°) impact.

As demonstrated by the simulation results for the same impact angle, the discrepancy in damage caused by oblique impact is predominantly attributable to the normal load component. Consequently, the 15° Bouligand structure was the sole structure selected for testing. The test arrangement is detailed in Table 6.

As the impact angle varies from 0° to 60°, the peak contact forces recorded in Figure 9c are 110.1 N, 80.6 N, 62.7 N, and 39.1 N, respectively. In the context of impact energy conditions that are identical, a negative correlation is exhibited by the peak contact force and the impact angle. As the impact angle increases, the energy absorbed by the laminate gradually decreases, as demonstrated in Figure 9d. For the same impact energy, variations in absorbed energy require analysis in conjunction with energy dissipation patterns (Figure 9f). The friction force curve (Figure 9e) indicates a positive correlation between impact angle and peak friction force. The dissipation of friction energy during the impact process is illustrated in Figure 9f, where the values of 3.120 × 10^−2^ J, 6.123 × 10^−1^ J, 9.724 × 10^−1^ J, and 1.278 J are observed, respectively.

Increases in helix angle have been shown to result in elevated levels of both the final peak friction force and the friction force time history, consequently leading to enhanced friction energy dissipation. Consequently, the energy absorbed by the specimen exhibits a difference and is negatively correlated with the impact angle, thereby reducing the overall structural damage.

**Figure 12:**
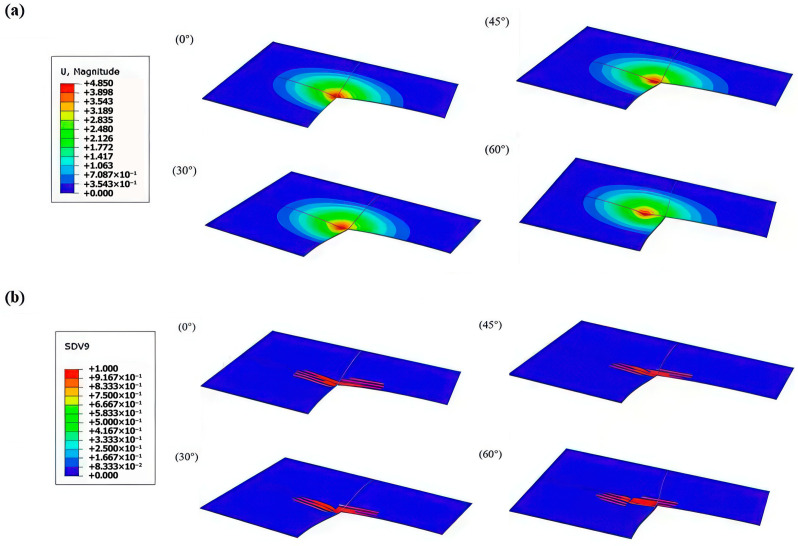
Impact response of the 3DPBL with a 15° helix angle under different impact angles. (**a**) Damage morphology. (**b**) Distribution of failed elements.

As illustrated in Figure 12, the 15° 3DPBL maintains a fundamental indentation mode characterized by plastic deformation once the normal load component exceeds the material’s yield strength. With increasing impact angles, the indentation center shifts laterally due to heightened tangential forces, while the maximum penetration depth exhibits a non-linear decrease—a trend that accelerates at higher obliquity.

This behavior stems from the discrete roles of the force components: the normal force governs depth, whereas the tangential force dictates the extent of the damage area, becoming the dominant factor beyond a 45° threshold. Kinematically, the oblique trajectory of the impactor engages a broader volume of structural elements and expands the contact area, significantly enhancing both element-level deformation and frictional energy dissipation. Consequently, the overall damage severity decreases at steeper impact angles, as the sacrificial deformation of the upper layers effectively shields the lower structure from significant damage.

**Figure 13:**
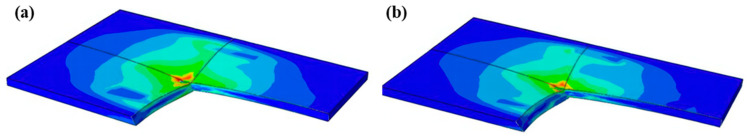
Comparison of internal stress fields: cross-sectional von Mises stress contours of the (**a**) 0° baseline and (**b**) 15° Bouligand layups subjected to a 30° oblique impact. Red regions denote areas of maximum stress concentration and potential material failure, while blue regions indicate low stress levels.

To further elucidate the energy dissipation mechanisms under oblique impact, Figure 13 compares the cross-sectional stress distributions of the 0° (Figure 13a) baseline and the 15° (Figure 13b) Bouligand laminates subjected to a 30° impact angle. Although both specimens exhibit comparable macroscopic indentations, their internal stress fields differ significantly. In the 0° layup, stress propagates preferentially along the principal fiber axes, resulting in a deeper through-thickness penetration of the high-stress zone. Conversely, the 15° helicoidal layup exhibits a broader and more uniform lateral stress dispersion pattern. The continuous inter-ply rotation progressively redirects the impact load, effectively converting localized normal penetration into extensive in-plane stress diffusion. This substantiates that optimizing the printing orientation fundamentally enhances internal load-bearing efficiency and preserves the integrity of the underlying structure.

During the occurrence of impactor slippage, internal elements within the 3DPBL undergo stress loading, with buckling phenomena propagating along the contact interface. The augmentation in the quantity of deformed elements has been demonstrated to engender a substantial enhancement in the efficiency of kinetic energy absorption. Concurrently, as the motion trajectory shifts, the increased contact area between the impactor and specimen leads to elevated friction energy dissipation, resulting in a nonlinear decrease in impact load per unit area. The combined effect of these two mechanisms effectively promotes impactor kinetic energy attenuation, thereby significantly delaying the progression of material damage. Subsequent analysis has indicated that the dissipation of impact energy is primarily attributable to plastic deformation in upper structural elements, thereby ensuring the effective preservation of the relative integrity of the underlying structure.

## 5. Conclusions

The 3DPBLs offer a promising pathway to overcome the limitations of traditional homogeneous materials in dynamic protection applications. To explore the potential of bio-inspired solutions, this study systematically investigated the mechanical response, damage characteristics, and energy dissipation mechanisms of the 3DPBLs under low-velocity oblique impact loading. Drop-weight impact tests were conducted on 3DPBLs with helix angles of 0°, 7.5°, 15°, 60°, and 90° to analyze their mechanical behavior and identify the optimal helix angle. Subsequently, a finite element model for normal low-velocity impact was established by implementing a sudden degradation constitutive model and the 3D-Hashin damage initiation criterion via a VUMAT subroutine. Following experimental validation, the model was extended to simulate inclined impact scenarios, facilitating a detailed analysis of the structural response and energy dissipation mechanisms under varying impact angles. The primary conclusions are summarized as follows:

Optimization of Helix Angle: The helix angle is identified as a critical determinant of impact resistance in 3DPBLs. The 15° configuration exhibited superior performance, yielding a peak contact force 20% higher than the 0° specimen and 10–15% higher than other tested angles (0°, 7.5°, 60°, and 90°).

Angle-Dependent Mechanical Response: The mechanical behavior shows a pronounced dependency on impact obliquity. Under normal impact, the structure relies on high fracture toughness to resist loads. Conversely, under oblique impact, the anisotropy and helical architecture facilitate interlaminar slip and relative deformation, effectively mitigating force transmission and dispersing impact energy.

Distinct Damage Mechanisms: Damage morphology varies significantly with impact angle. While both loading conditions induce through-thickness cracks, normal impact is characterized by interlaminar delamination and brittle propagation. In contrast, oblique impact results in larger damage areas but shallower indentation depths. This is attributed to the complex helical layup, which creates a tortuous crack propagation path, thereby suppressing vertical crack growth and increasing the energy required for failure. This mechanism significantly increases fracture tortuosity, thereby extending the crack paths and effectively mitigating brittle failure.

Energy Dissipation Modes: The dominant energy dissipation mechanisms shift with the loading angle. Perpendicular impacts dissipate energy primarily through elastic-plastic deformation during crack propagation. However, oblique impacts rely more heavily on interlaminar slip and tensile stretching. This shift in mechanism underpins the structure’s superior adaptability to varying impact directions.

Implications for Design: The analysis confirms that 3DPBLs possess a highly tunable mechanical response. By optimizing the interlayer angle, the structure demonstrates exceptional energy absorption and damage suppression, particularly under oblique loading. This adaptability highlights the potential of 3DPBLs for advanced protective applications subjected to multi-directional threats.

These findings highlight the unique tunable damage tolerance characteristics of 3DPBLs and underscore the necessity of considering impact directionality in the design of advanced impact-resistant composite structures.

It should be noted that the current study focuses specifically on the impact resistance and crack propagation mechanisms of brittle materials (PLA) within the bio-inspired spiral architecture. While ductile materials such as ABS or PC may exhibit different energy absorption characteristics, the use of a brittle matrix allows for a clearer observation of how the structural geometry guides fracture paths. Due to the experimental timeline, testing of ductile or impact-modified filaments was not included in this work but remains a critical direction for our future comparative research.

## Figures and Tables

**Table 1:** Material Parameters of the PLA 3DPBL.

Materials	Properties	Parameters
PLA Single-Layer Board Properties	Density (kg/m^3^)	ρ = 1220
	Young’s Modulus (GPa)	E_11_ = 3.6; E_22_ = E_33_ = 3.3; G_12_ = G_13_ = 1.25; G_23_ = 1.14
	Poisson’s Ratio (υ)	υ_12_ = υ_13_ = 0.3; υ_23_ = 0.35
	Strength (MPa)	X^T^ = 33; X^C^ = 50; Y^T^ = 25; Y^C^ = 35;
		S_12_ = S_13_ = S_23_ = 23
Interlayer Properties	Fracture Energy (N/mm)	G_ft_ = 1.0; G_fc_ = 2.8; G_mt_ = 0.4; G_mc_ = 1.8
	Strength (MPa)	N = S = T = 30
	Fracture Energy (N/mm)	G_Ic_ = 0.35; G_IIc_ = G_IIIc_ = 0.75
	Correlation Coefficient (η)	η = 1.45

**Table 2:** Mean and standard deviation of peak contact forces from triplicate tests.

Angle (°)	Group 1 (N)	Group 2 (N)	Group 3 (N)	Mean (N)	Standard Deviation (%)
0°	79.80	74.42	84.27	79.50	6.21
7.5°	99.86	93.27	103.21	98.78	5.14
15°	116.49	123.38	109.69	116.52	5.87
60°	109.19	113.21	104.24	108.88	4.13
90°	110.22	120.51	104.59	111.77	7.24

**Table 3:** Cohesive model material parameters.

Strength		Fracture Energy		Correlation Coefficient
**N**	30 MPa	G_I_	3.5 × 10^2^ J/m^2^	1.45
**S**	30 MPa	G_II_	7.5 × 10^2^ J/m^2^	
**T**	30 MPa	G_III_	7.5 × 10^2^ J/m^2^	

**Table 4:** Results and relative errors of simulated and experimental peak contact force Fmax.

Helix Angle α	Simulation Value	Experimental Value	Relative Error
**0°**	75.20 N	79.80 N	3.0%
**7.5°**	91.06 N	99.86 N	4.6%
**15°**	110.10 N	116.49 N	2.8%
**60°**	101.96 N	109.19 N	3.4%
**90°**	101.93 N	110.22 N	3.9%

**Table 5:** Design of inclined impact numerical simulation plan I.

Impact Angle θ (°)	Impact Energy Ea (J)	Helix Angle α (°)
45/0	2.5	7.5/15/60/90

**Table 6:** Experiment II protocol design.

Impact Angle θ (°)	Impact Energy Ea (J)	Helix Angle α (°)
0/30/45/60	2.5	15

## Data Availability

The original contributions presented in this study are included in the article. Further inquiries can be directed to the corresponding author.
